# How does Working Memory Promote Traces in Episodic Memory?

**DOI:** 10.5334/joc.245

**Published:** 2023-01-11

**Authors:** Vanessa M. Loaiza, Anne-Laure Oftinger, Valérie Camos

**Affiliations:** 1 Department of Psychology, University of Essex, GB; 2 Department of Psychology, University of Fribourg, CH

**Keywords:** working memory, refreshing, covert retrieval, episodic memory

## Abstract

A longstanding research question in cognitive psychology concerns how the underlying mechanisms of working memory impact long-term episodic memory. In this series of six experiments, we manipulated three different factors within a complex span task that interleaves memoranda and distractors to investigate the contribution of these factors to the creation of episodic traces: (1) the cognitive load of processing the distractors, (2) the number of distractors, and (3) the free time following the distractors. All three factors have been identified in the prior literature as important to maintenance in working memory and, consequently, later retrieval from episodic memory. Thus, it is important to understand their unique and joint effects to the long-term durability of memory traces. Across six experiments, delayed recall (i.e., episodic memory) of the items studied during the complex span tasks (i.e., working memory) was best accounted for by accumulated free time, whereas the effects of cognitive load and number of distractors were inconsistent or negligible. These results conflict with prior work suggesting that cognitive load and the number of distractors impact episodic memory. However, the current results replicate and extend those suggesting that time spent processing items in working memory promotes the creation of episodic memory traces.

Working memory (WM) is the system that maintains a limited amount of information for ongoing cognition. There has been renewed interest in how WM processes promote long-term retention of traces in episodic memory (EM; e.g., Camos & Portrat, 2015; Jarjat et al., 2018, 2020; Loaiza & McCabe, 2012, 2013; McCabe, 2008; Rose et al., 2014; Souza & Oberauer, 2017). WM is often tested using complex span tasks that alternate memoranda (e.g., words) with distractors (e.g., parity decisions of whether a digit is even or not). The current experiments aimed at better understanding how information briefly held in WM is more durably available in EM by orthogonally varying three different factors in WM and assessing their impact on a delayed test of EM. We discuss the prior research on each factor before introducing the current experiments.

## The Cognitive Load of Distractors in Working Memory

Extensive work has shown that increasing the cognitive load of distractors (e.g., making parity decisions at a fast versus slow pace) strongly impairs immediate recall from WM (e.g., Barrouillet & Camos, 2012; Barrouillet et al., 2007, 2004, 2011). According to the time-based-resource sharing (TBRS) model (Barrouillet & Camos, 2015), this cognitive load effect occurs due to reduced opportunity to keep memoranda active in WM via refreshing, or focused attention to recently active memory traces (see Camos et al., 2018 for review), which likewise impacts EM. Camos and Portrat (2015) tested this hypothesis by varying the cognitive load of complex span distractors via their difficulty (serial response time, SRT, versus parity; Experiment 1) or pace of presentation (slow versus fast pace; Experiment 2). Consistent with prior work, WM recall was reduced for high versus low cognitive loads, and this detrimental effect of cognitive load was also evident EM recall. Thus, varying cognitive load may vary the opportunity to engage in refreshing that impacts both the short-term and long-term durability of memory traces.

## The Number of Distractors in Working Memory

Other work has focused on the long-term impact of the number of distractors interleaving the memoranda in a complex span task. According to the covert retrieval model (McCabe, 2008), covert retrieval must recover the displaced memoranda after processing distractors, in turn affording covert retrieval opportunities that promote subsequent EM. McCabe (2008) tested this hypothesis by comparing immediate and delayed recall of items presented during simple and complex span tasks. During simple span tasks, only memoranda are presented successively for recall without distractors. Although immediate recall was unsurprisingly greater for simple than complex span, the reverse was true during delayed recall. Loaiza and McCabe (2013) further reported that recall from EM increased with the number of distractors preceding each memorandum in a complex span task. Thus, increasing distractors in WM may inadvertently promote retrieval from EM.

## The Free Time Following Distractors in Working Memory

According to the processing time hypothesis (Jarjat et al., 2018, 2020; Souza & Oberauer, 2017), the longer the memoranda are processed in WM, the more likely they are to be retrieved later on from EM. Souza and Oberauer (2017) tested this idea by intermixing trials of complex span, simple span, and slow span for immediate and delayed recall. Slow span is more akin to simple span in that the memoranda successively appear, but with free time of equal duration to the distractors interleaving the memoranda during complex span. Souza and Oberauer reported that delayed recall was greater for the slow span versus the complex and simple span items. Thus, uninterrupted free time may reinforce WM traces to make them more available for later recall from EM. The benefit of free time may be due to greater opportunities to employ elaborative strategies and/or enhanced resources to encode or consolidate the memory traces (see Mızrak & Oberauer, 2021; Popov & Reder, 2020; Souza & Oberauer, 2017 for further discussion).

## How Do These Factors Interact?

Jarjat and colleagues (2018, 2020) attempted to dissociate two of these factors by orthogonally manipulating the cognitive load (reading digits at a slow or fast pace) and number of distractors (2 or 8 digits) during complex span. Consistent with prior work, they observed negative effects of cognitive load and positive effects of number of distractors, with no interaction. The authors considered whether these effects could be accounted for by a more parsimonious explanation: A lower cognitive load, such as through a slow pace, and a greater number of distractors, both serve to prolong the total time the memoranda are processed in WM. Thus, the effects of cognitive load and distractors could be better understood as a cumulative impact of free time on EM, consistent with Souza and Oberauer (2017). Indeed, when Jarjat and colleagues analyzed delayed recall as a function of the estimated free time (i.e., the time remaining after processing distractors) that had accumulated for each item across a trial, they showed a logarithmic relationship: The impact of accumulated free time on delayed recall was strongest earlier on but less beneficial as time progressed. These results reinforced the notion that the time that memoranda spend in WM promotes their long-term retention.

## Current Experiments

We designed six experiments to disentangle how these three factors (i.e., cognitive load of distractors, number of distractors, and free time following distractors) in WM may uniquely and jointly impact later retrieval from EM. All of the experiments had the same basic procedure: a complex span task presented a series of distractors that followed each of four to-be-remembered words to immediately recall in their original order of presentation (i.e., immediate serial recall; WM). Later on, participants recalled the words regardless to their original serial position (i.e., delayed free recall; EM). Most importantly, we varied either the cognitive load of the distractors (Experiments 1–6), the number of distractors following each to-be-remembered word (Experiments 1–4), and/or the free time following each distractor (Experiments 5–6; see Table 1). The precise details and justifications of the methodological differences of the manipulations for each factor across experiments are explained further on. Unfortunately, it is not possible to orthogonally manipulate all three variables in the same experiment. In short, varying two factors (e.g., cognitive load and number of distractors) necessarily varies the third factor (e.g., free time) in a way that cannot be disambiguated from one or both of the other factors. Thus, we carefully designed our experiments with this unavoidable drawback in mind in order to investigate the contribution of each factor to EM. Finally, consistent with prior work, the accumulated free time resulting from the different experimental manipulations was computed.

**Table 1:** Overall summary of the current experiments.


METHODOLOGICAL DETAILS		EXPERIMENT					

		1	2	3	4	5	6

Participants	Mean age (SD)	21.29 (1.85)	20.33 (1.66)	21.79 (2.25)	21.63 (2.08)	24.42 (4.64)	21.13 (1.75)

	Experiment language	Italian	Italian	Italian	English	English	Italian

Design	Overall design	2 (CL) × 2 (# Dist)	2 (CL) × 2 (# Dist)	2 (CL) × 2 (# Dist)	2 (CL) × 2 (# Dist)	2 (CL) × 2 (FT)	One-way: High CL/Low FT Low CL/High FT High CL/High FT

	CL (low vs. high)	Location vs. Parity	Slow vs. Fast Pace	SRT vs. Parity	SRT vs. Parity	Slow vs. Fast pace	Slow vs. Fast pace

	# Dist (low vs. high)	3 vs. 6	3 vs. 6	3 vs. 6	1 vs. 3	–	–

	FT (low vs. high)	–	–	–	–	Short vs. Long	Short vs. Long

Method	# Dist after each word	3 or 6	3 or 6	3 or 6	1 or 3	2, 4, or 8	3 or 6

	Pace of each distractor	700 ms	1125 or 600 ms	700 ms	700 ms	750 ms	600 ms

	FT after each distractor	300 ms	375 or 200 ms	300 ms	300 ms	500 or 250 ms	400 or 200 ms

	Total FT/studied word	900 or 1800 ms	from 600 to 2250 ms	900 or 1800 ms	300 or 900 ms	2000 or 1000 ms	1200 or 600 ms

	Immediate recall method	Typed	Typed	Typed	Aloud	Aloud	Typed


*Note*: All design factors manipulated within-subjects, and blocked and counterbalanced across participants. CL = cognitive load, # Dist = number of distractors, FT = free time, SRT = serial reaction time.

Our primary dependent variable was delayed recall, both overall and conditionalized on accurate immediate recall to ensure that any patterns in delayed recall were not simply an artifact of differential rates of immediate recall across conditions. We expected to both replicate the effects of each of the three factors on EM and extend these results to elucidate how the factors may interact with each other in their contributions to EM.

## Method

### Participants

We collected datasets from 24 unique participants per experiment. This planned sample size was determined from previous similar experiments demonstrating significant effects of cognitive load and distractors on delayed recall (Camos & Portrat, 2015; McCabe, 2008). All of the participants provided informed consent before beginning and were debriefed at the end of the experiment. All participants were recruited from the authors’ university subject pools in exchange for partial course credit or monetary compensation. The Ethics committees of the Universities of Fribourg (Experiments 1–3 and 6) and Essex (Experiments 4–5) approved the ethics applications for the experiments in accordance with the tenets of the Declaration of Helsinki.

### Materials

Experiments 1–3 and 6 were programmed in E-prime (Psychology Software Tools, Inc., 2012), and Experiments 4–5 were programmed in MATLAB with Psychtoolbox (Brainard, 1997; Kleiner et al., 2007). The memoranda for Experiments 1–3 and 6 were 128 highly-frequent Italian nouns drawn from the Lexvar database (Barca et al., 2002). An additional 16 words were used for the examples. The critical memoranda were two syllables long and ranged between 4–8 letters (*M* = 4.95, *SD* = 0.90). They were randomly arranged into separate sets that were counterbalanced across participants and drawn randomly without replacement from the sets. The memoranda for Experiments 4–5 comprised 154 highly frequent English nouns drawn from the English Lexicon database (Balota et al., 2007). The memoranda were between 1–2 syllables long (*M* = 1.47, *SD* = 0.50) and ranged between 4–8 letters (*M* = 5.35, *SD* = 1.29). They were randomly drawn without replacement for each participant.

### Procedure

The six experiments all followed the same format, with the differences between them outlined in Table 1. The whole experiment was completed in the participants’ native language, and participants were tested individually with an experimenter present. There were two main phases: a practice phase and a critical phase.

**Practice phase.** Participants first began with a practice phase of what would eventually be the distractors presented during the critical phase of the experiments. There was a corresponding practice phase for each of the cognitive load conditions of the critical phase, described in detail in the next subsection. Each practice phase comprised 15 trials, with 4 trials used as examples during the instructions. Participants were required to reach an 85% criterion for each practice phase, and they repeated the practice phase until they achieved this criterion. Participants responded using one of two designated keys on the keyboard as well as an aloud response of “yes” or “no” depending on the nature of the decision for the task.

**Critical phase.** After completing the practice phase, the critical phase of the experiment began, wherein participants completed four (Experiments 1–5) or three (Experiment 6) blocks of the complex span task, with delayed free recall following each block. Each block represented a different counterbalanced condition of the manipulated factors of the experiment. Each block comprised 8 trials of 4 to-be-remembered words, with 2 practice trials preceding each block.

The format of the trials was similar across experiments: A fixation cross appeared at the center of the screen for 1500 ms, followed by the first to-be-remembered word presented for 1000 ms (500 ms interstimulus interval, ISI). Participants read the words aloud (Experiments 1–3 and 6) or silently because they were required to repeat “the” continuously throughout the trial (Experiments 4–5). The distractors followed each word, the nature of which depended on the experiment manipulation and counterbalance order of the blocks. In Experiment 1, a square with a digit (between 1 and 9) inside it was presented, and participants decided whether the square was in the upper part of the screen or not (location) or whether the digit in the square was even or not (parity). In Experiments 2, 5, and 6, participants made parity decisions on digits that were presented at the center of the screen at a slow or fast pace. In Experiments 3 and 4, participants either responded to digits presented at the center of the screen by simply pressing the spacebar as they appeared (serial reaction task, SRT) or making a parity decision. In Experiments 1–3 and 6, participants responded using the keyboard while also saying “yes” or “no” aloud, whereas participants in Experiments 4–5 responded only with the keyboard while repeating “the” continuously throughout the trial. Interleaving the words, there were either 3 or 6 distractors (Experiments 1–3 and 6), 1 or 3 distractors (Experiment 4), and 2, 4, or 8 distractors (Experiment 5). Note that, as will be clear further on in the Results section, the point of these methodological differences between experiments regarding the number of and way in which the distractors were presented was to illicit stronger effects of cognitive load and number of distractors. At the end of the trial, participants were prompted to recall the words in their original serial order by either typing them with their responses echoed on screen (Experiments 1–3 and 6) or out loud (Experiments 4–5), the latter of which were audio-recorded and transcribed offline.

After completing each block, participants completed an unrelated task wherein they silently added a series of sequentially presented two-digit numbers (e.g., 45 + 22 = ?) at a self-paced rate (250 ms ISI). After 2 min, participants received instructions for the delayed recall test. Following prior work, participants were instructed to recall as many of the words from the previous block as possible, without regard to their original order of presentation, by typing their responses using the keyboard and their responses were echoed on screen. In these instances where participants typed their immediate recall (Experiments 1–3 and 6) and delayed recall (all experiments), the rare instances of typos were corrected if they were not ambiguous (e.g., a common typo of “reciept” was corrected to “receipt”, but “horm” was not corrected because it could be corrected as “harm” or “horn”; see Loaiza & Lavilla, 2021 for similar).

### Analysis

All practice trials were excluded from analysis, and the accuracy and response times (RTs) to the distractors that were faster than 100 ms or timeouts (6% of the data) were also excluded from the distractor analysis. The data of one block of one participant in Experiment 5 were missing due to experiment failure and excluded from analysis. We used the BayesFactor package (Morey & Rouder, 2015) with its default settings for analysis.

## Results and Discussion

### Complex Span Performance

We first report performance on the distractors (response times, RTs) and immediate recall (serial scoring, i.e., correctly recalling a word in its original serial position) during the complex span task (Tables 2 and 3).^1^ Experiments 1–4 replicated prior work that increasing cognitive load impacts RTs and immediate recall, ensuring that the cognitive load manipulations were sound and thus should also affect delayed recall. There was a cognitive load effect on distractor RTs but not on immediate recall in Experiment 5, and there were no cognitive load effects in Experiment 6. Manipulating free time in these experiments may have mitigated the cognitive load effects.

**Table 2:** Mean (and standard deviations) of distractor response times (RTs, in ms) and immediate recall (proportion correct) as a function of cognitive load (CL) and the second manipulated factor (either distractors or free time) in each experiment.


EXP.	SECOND FACTOR		RESPONSE TIMES (RTS, MS)		IMMEDIATE RECALL – SERIAL SCORING	
	
			LOW CL	HIGH CL	LOW CL	HIGH CL

1	Distractors	Low	403.56 (56.87)	549.62 (36.20)	0.73 (0.14)	0.65 (0.18)

		High	378.02 (42.75)	547.93 (26.39)	0.72 (0.21)	0.61 (0.15)

2	Distractors	Low	605.21 (83.19)	514.43 (28.04)	0.79 (0.15)	0.68 (0.18)

		High	606.76 (74.98)	521.82 (34.13)	0.78 (0.16)	0.62 (0.19)

3	Distractors	Low	285.21 (53.03)	542.99 (42.83)	0.86 (0.12)	0.70 (0.15)

		High	298.26 (56.26)	540.41 (42.03)	0.90 (0.12)	0.63 (0.18)

4	Distractors	Low	576.51 (108.28)	709.07 (72.06)	0.48 (0.24)	0.38 (0.24)

		High	587.25 (130.59)	686.73 (63.25)	0.50 (0.24)	0.33 (0.23)

5	Free time	Low	737.75 (78.22)	717.88 (64.77)	0.44 (0.22)	0.45 (0.18)

		High	736.01 (77.28)	707.86 (60.36)	0.47 (0.23)	0.46 (0.26)

6	Free time	Low	–	492.32 (22.20)	–	0.69 (0.14)

		High	499.53 (38.66)	497.38 (24.00)	0.68 (0.17)	0.62 (0.19)


*Note*: Exp. = experiment, CL = cognitive load.

**Table 3:** Results of the BANOVAs for each experiment.


EXP.	DESIGN	MEASURE	MAIN EFFECT OF FACTOR 1	MAIN EFFECT OF FACTOR 2	BOTH MAIN EFFECTS	MAIN EFFECTS + INTERACTION

1	2 (CL: location, parity) × 2 (Distractors: 3, 6)	Distractor RTs	1.52 × 10^36^	0.27	2.65 × 10^36^	**3.53 × 10^36^**

		Immediate serial recall	**134.32**	0.35	53.72	19.42

		Delayed recall overall	0.32	0.87	0.30	0.14

		Delayed recall conditionalized	0.23	1.15	0.27	0.09

2	2 (CL: slow, fast pace) × 2 (Distractors: 3, 6)	Distractor RTs	**1.00 ×10^13^**	0.22	2.50 × 10^12^	7.55 × 10^11^

		Immediate serial recall	**2860.07**	0.40	1453.28	603.75

		Delayed recall overall	**47.94**	0.22	10.81	3.14

		Delayed recall conditionalized	1.64	0.22	0.38	0.11

3	2 (CL: SRT, parity) × 2 (Distractors: 3, 6)	Distractor RTs	**6.29 ×10^47^**	0.22	1.75 × 10^47^	8.12 × 10^46^

		Immediate recall	**2.36 × 10^10^**	0.23	6.21 × 10^9^	1.83 × 10^10^

		Delayed recall overall	**120.08**	0.29	38.02	11.24

		Delayed recall conditionalized	1.94	0.43	0.90	0.36

4	2 (CL: SRT, parity) × 2 (Distractors: 1, 3)	Distractor RTs	**3.44 ×10^8^**	0.21	7.61 × 10^7^	4.72 × 10^7^

		Immediate serial recall	**416.07**	0.23	102.49	50.59

		Delayed recall overall	2.98	0.58	1.94	0.69

		Delayed recall conditionalized	0.30	**8.83**	2.87	0.83

5	2 (CL: slow, fast pace) × 2 (FT: short, long)	Distractor RTs	**25.56**	0.28	7.73	2.61

		Immediate serial recall	0.22	0.24	0.06	0.02

		Delayed recall overall	0.43	0.43	0.19	0.06

		Delayed recall conditionalized	1.98	0.50	1.08	0.32

6	One-way (High CL/Low FT, Low CL/High FT, High CL/High FT)	Distractor RTs	0.16	–	–	–

		Immediate serial recall	0.67	–	–	–

		Delayed recall overall	0.21	–	–	–

		Delayed recall conditionalized	0.22	–	–	–


*Note*: All models include participant as a random effect. The Bayes factor (BF) refers to the evidence for the alternative model (BF_10_) for each effect (shown in the different columns) relative to the null model (i.e., a model that includes only a random effect of participant). The best model in favor of the effect is shown in boldface in each row for each experiment/measure and is underlined when the BF for the best model relative to the next-best model exceeds 3. BANOVA = Bayesian analysis of variance, Exp. = experiment, CL = cognitive load, FT = free time, RTs = response times.

### Delayed Recall Performance

The hypotheses concerned (overall and conditionalized) delayed recall performance. The results are split according to the manipulations of cognitive load and number of distractors (Experiments 1–4) and cognitive load and free time (Experiments 5–6). Note that we did not go onto manipulate free time and number of distractors in further experiments due to the overall pattern of results indicating that this would not be worth pursuing.

**Figure 1:**
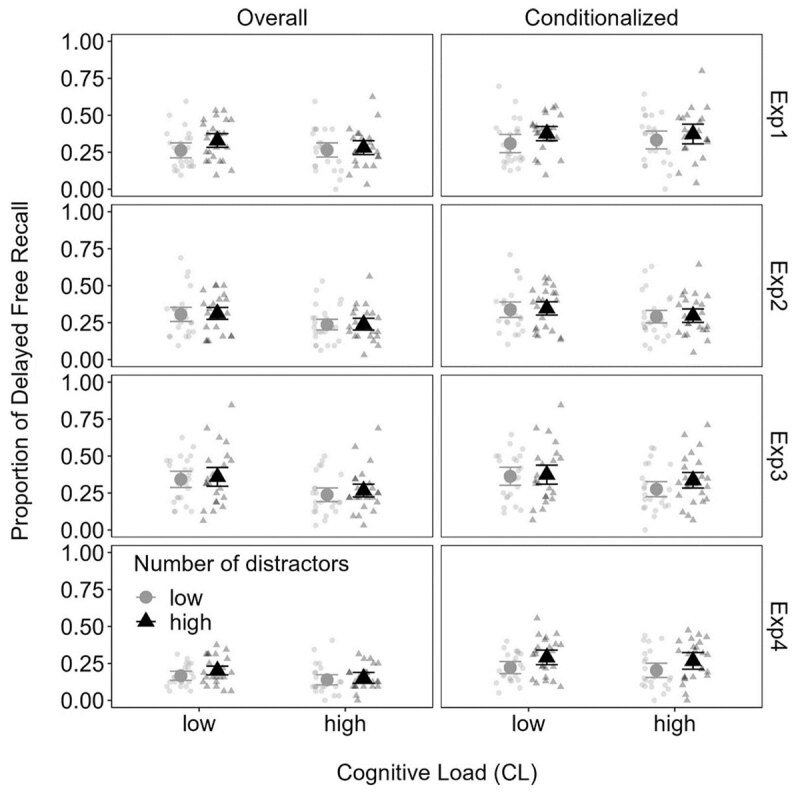
Mean overall and conditionalized delayed recall (and 95% within-subjects confidence intervals) as a function of cognitive load and number of distractors in Experiments 1–4.

**Experiments 1–4.** In Experiment 1, there was substantial evidence against a cognitive load effect (Figure 1, Table 3). In Experiments 2 to 3, a more dramatic contrast of cognitive load conditions yielded stronger effects on overall delayed recall, but only ambiguous effects on conditionalized delayed recall. This is problematic because the cognitive load effect cannot be disambiguated from an artifact of simply having more prior retrieval practice for the low cognitive load items. Moreover, the effect the number of distractors was also either ambiguous or favored the null in Experiments 1 to 3 for both types of delayed recall. Thus far, these results contradict the notion that cognitive load and number of distractors moderate retrieval from EM.

Experiment 4 implemented a more strenuous constraint on articulatory rehearsal by requiring participants to continuously repeat “the” throughout the trial. Furthermore, we changed the manipulation of distractors from 3 and 6 in Experiments 1–3 to 1 and 3 in Experiment 4 in order to more closely align to prior work (Loaiza & McCabe, 2013). Although there was an even stronger cognitive load effect on immediate recall, there were no such substantial effects on delayed recall. There was also no evidence for an effect of the number of distractors on overall delayed recall, but there was a substantial effect in conditionalized delayed recall. At first glance, this latter result suggests that there may be diminishing returns of the number of distractors on delayed recall. Perhaps up to 3 distractors after each word is sufficient to yield a benefit to later delayed recall, with no difference emerging when comparing 3 to 6 distractors in Experiments 1–3. However, Jarjat and colleagues (2018) observed a benefit of 8 versus 2 distractors, and so it may be instead that the stronger articulatory suppression in Experiment 4 strengthened the impact of the number of distractors on conditionalized delayed recall.

**Figure 2:**
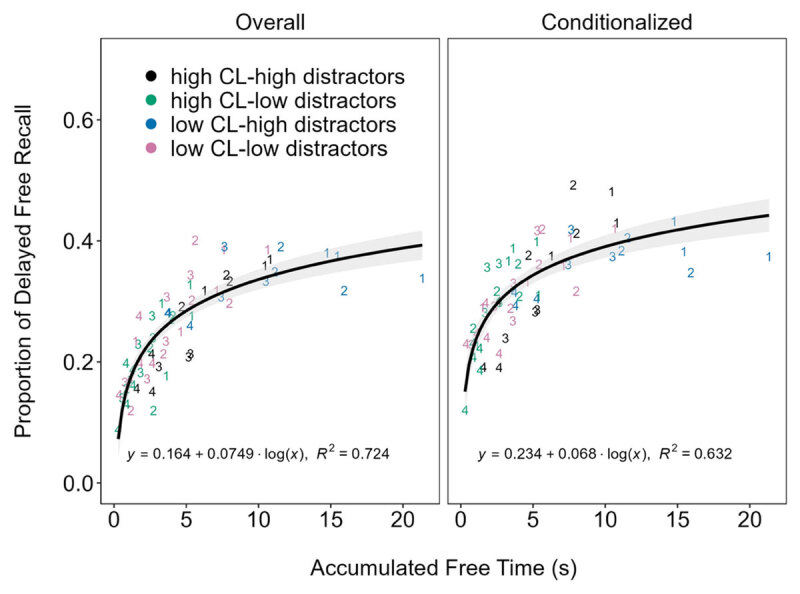
Delayed recall (both overall and conditionalized) as a function of accumulated free time at each serial position (labeled 1–4) and each combination of cognitive load (CL) and number of distractors conditions in Experiments 1–4. See online article for a color version of this figure.

Finally, we plotted delayed recall at each serial position as a function of accumulated free time (Figure 2). That is, we subtracted the distractor keypress decision RT from the total time allotted for each distractor, and then summed this remainder time across the distractors for each word according to its position in the trial to approximate the average accumulated free time each serial position received across the trials. Our results showed a logarithmic relationship between accumulated free time and delayed recall, both overall (BF_10_ = 5.78 × 10^15^) and conditionalized (BF_10_ = 2.32 × 10^12^). Further similar to Jarjat and colleagues (2018), the logarithmic model was overwhelmingly preferred (BFs > 297,000) to the linear model for both overall (BF_10_ = 1.38 × 10^10^) and conditionalized (BF_10_ = 7.79 × 10^6^) delayed recall. Thus, the benefits of free time were less pronounced as accumulated free time progressed (e.g., an additional 1s of free time yielded a stronger benefit for words that accumulated 1s of free time at that point compared to 10s).

**Figure 3:**
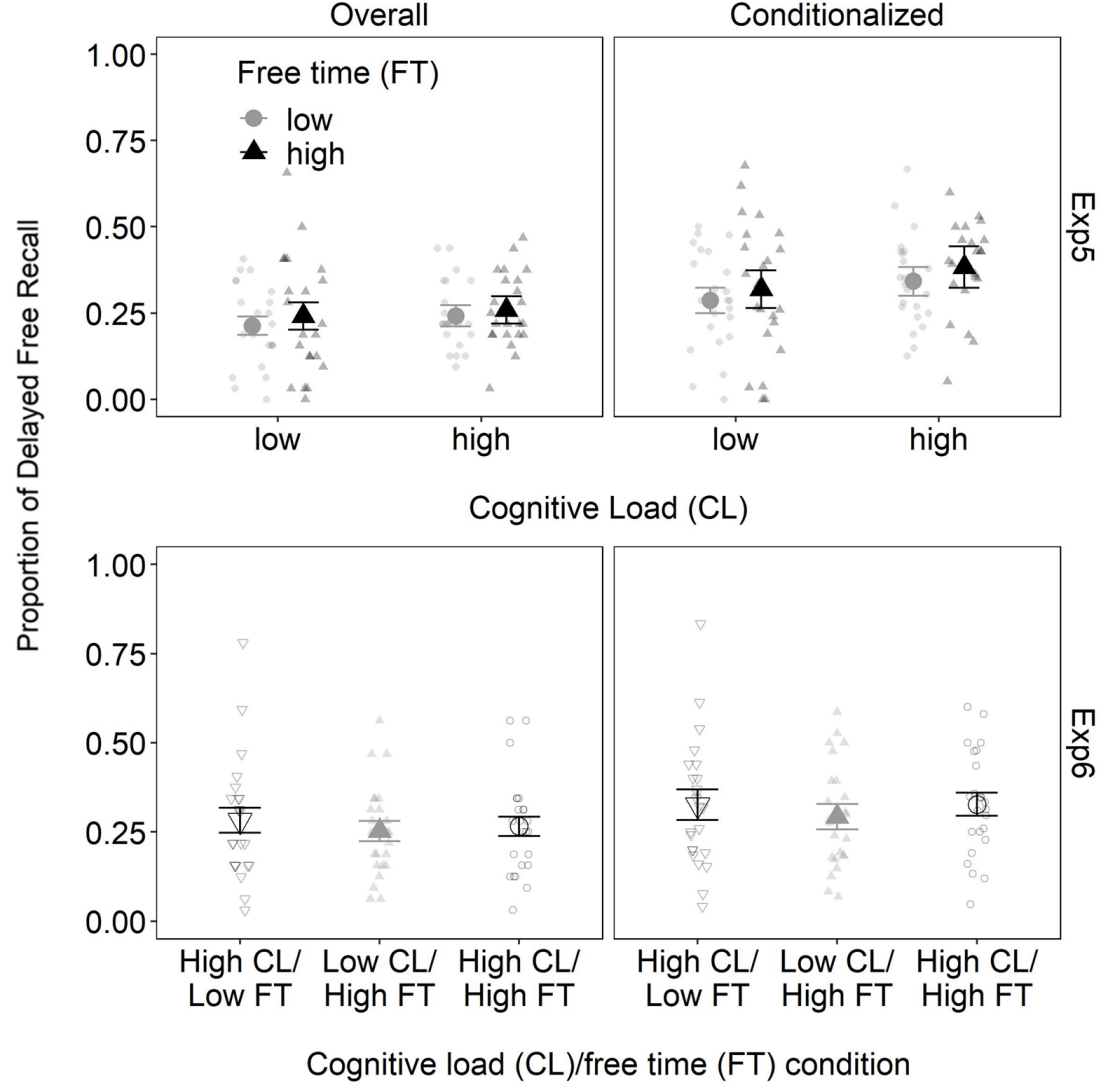
Mean overall and conditionalized delayed recall (and 95% within-subjects confidence intervals) as a function of cognitive load and free time in Experiments 5–6.

**Experiments 5–6.** Experiments 5–6 assessed the role of free time in WM for long-term retention alongside cognitive load (Figure 3, Table 3). There were no cognitive load effects on delayed recall in either experiment. This may be expected given the lack of cognitive load effects on immediate serial recall. However, to our surprise given the previous correlational relationship, there was no evidence of free time effects on delayed recall either.

**Figure 4:**
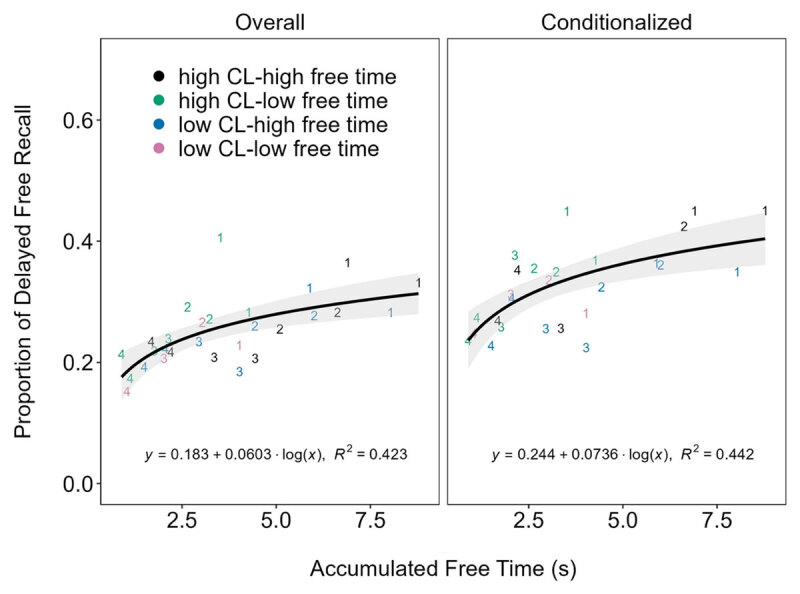
Delayed recall (both overall and conditionalized) as a function of accumulated free time at each serial position (labeled 1–4) and each combination of cognitive load (CL) and free time conditions in Experiments 5–6. See online article for a color version of this figure.

We once again considered the relationship between accumulated free time and delayed recall in Experiments 5–6 (Figure 4). The evidence for the logarithmic relationship was still strong but greatly reduced for both overall (BF_10_ = 127) and conditionalized (BF_10_ = 183) delayed recall. Furthermore, the logarithmic models were only ambiguously preferred (BFs = 1–1.6) to the linear models of overall (BF_10_ = 79) and conditionalized (BF_10_ = 183) delayed recall.

## Conclusions

Across six experiments, delayed recall was best accounted for by accumulated free time, with inconsistent effects of the other manipulated factors. This latter finding contradicts prior work suggesting that cognitive load and number of distractors impacts EM (Camos & Portrat, 2015; Loaiza & McCabe, 2013), but the former is consistent with other work suggesting that accumulated free time in WM is correlated with EM (Jarjat et al., 2018, 2020). It is not possible to determine why there may have been negligible effects of cognitive load and number of distractors in the current work. Perhaps prior effects of these factors are simply better accounted for by a parsimonious explanation of free time, as other work has suggested (Jarjat et al., 2018; Souza & Oberauer, 2017). Accordingly, we encourage future research to focus on testing the different accounts for why free time in WM promotes traces in EM (e.g., Mızrak & Oberauer, 2021; Popov & Reder, 2020; Souza & Oberauer, 2017).

## Data Accessibility Statement

None of the experiments were pre-registered. The experimental materials, raw data, and analysis scripts for all the experiments are available at https://osf.io/dujhq.

## Footnotes

Further analyses of distractor accuracy and free scoring of immediate recall (i.e., correctly recalling a presented word regardless of its serial position) can be found on the OSF.
